# Socioeconomic inequalities in smoking habits are still increasing in Italy

**DOI:** 10.1186/1471-2458-14-879

**Published:** 2014-08-27

**Authors:** Giuseppe Verlato, Simone Accordini, Giang Nguyen, Pierpaolo Marchetti, Lucia Cazzoletti, Marcello Ferrari, Leonardo Antonicelli, Francesco Attena, Valeria Bellisario, Roberto Bono, Lamberto Briziarelli, Lucio Casali, Angelo Guido Corsico, Alessandro Fois, MariaGrazia Panico, Pavilio Piccioni, Pietro Pirina, Simona Villani, Gabriele Nicolini, Roberto de Marco

**Affiliations:** Unit of Epidemiology and Medical Statistics, Department of Public Health and Community Medicine, University of Verona, Verona, Italy; Department of Medicine, University of Verona, Verona, Italy; Allergy Unit, Ospedali Riuniti di Ancona, Ancona, Italy; Department of Public, Clinical and Preventive Medicine, II University of Naples, Naples, Italy; Department of Public Health and Pediatrics, University of Turin, Turin, Italy; Department of Hygiene, University of Perugia, Perugia, Italy; Department of Internal Medicine, Section of Respiratory Disease, University of Perugia, Perugia, Italy; Institute of Respiratory Diseases, IRCCS San Matteo, Pavia, Italy; Institute of Respiratory Diseases, University of Sassari, Sassari, Italy; Unit of Epidemiology, ASL Salerno 2, Salerno, Italy; Unit of Respiratory Medicine, National Health Service (CPA-ASL TO2), Turin, Italy; Department of Public Health, Experimental and Legal Medicine, University of Pavia, Pavia, Italy; Corporate Clinical Development, Chiesi Farmaceutici S.p.A, Parma, Italy; Sezione di Epidemiologia e Statisca Medica, Istituti Biologici 2B, Strada Le Grazie 8, 37134 Verona, Italy

**Keywords:** Smoking initiation, Smoking cessation, Trends, Socioeconomic status, Italy

## Abstract

**Background:**

Socioeconomic inequalities in smoking habits have stabilized in many Western countries. This study aimed at evaluating whether socioeconomic disparities in smoking habits are still enlarging in Italy and at comparing the impact of education and occupation.

**Methods:**

In the frame of the GEIRD study (Gene Environment Interactions in Respiratory Diseases) 10,494 subjects, randomly selected from the general population aged 20–44 years in seven Italian centres, answered a screening questionnaire between 2007 and 2010 (response percentage = 57.2%). In four centres a repeated cross-sectional survey was performed: smoking prevalence recorded in GEIRD was compared with prevalence recorded between 1998 and 2000 in the Italian Study of Asthma in Young Adults (ISAYA).

**Results:**

Current smoking was twice as prevalent in people with a primary/secondary school certificate (40-43%) compared with people with an academic degree (20%), and among unemployed and workmen (39%) compared with managers and clerks (20-22%). In multivariable analysis smoking habits were more affected by education level than by occupation. From the first to the second survey the prevalence of ever smokers markedly decreased among housewives, managers, businessmen and free-lancers, while ever smoking became even more common among unemployed (time-occupation interaction: p = 0.047). At variance, the increasing trend in smoking cessation was not modified by occupation.

**Conclusion:**

Smoking prevalence has declined in Italy during the last decade among the higher socioeconomic classes, but not among the lower. This enlarging socioeconomic inequality mainly reflects a different trend in smoking initiation.

## Background

The smoking epidemic is fading away in both sexes in most developed countries [1–4]. Not only smoking prevalence is shrinking, but also the average number of cigarettes consumed daily by people who still smoke [3, 5].

In this late stage of smoking epidemic the prevalence of current smoking generally becomes inversely related to socioeconomic status [6], usually evaluated by education level, occupation and income. Probability of smoking initiation is higher in people with low education while probability of quitting smoking is higher in highly educated people [7–12]. Educational inequalities in smoking habits affect both sexes in Northern Europe, while they are apparently restricted to men in Southern Europe [7]: however, recently the prevalence of current smokers has been reported to decrease with increasing educational level both in Italian men and women, although the trend was significant only in men [13]. As regards occupation, blue collars and unemployed have a higher risk of starting smoking and a lower risk of quitting than white collars [14–17]. In most European countries income is less important than either education [18, 19] or employment status [16] in predicting smoking habits. At variance, the magnitude of income- and education-related inequalities is similar among women in Southern Europe [18] and in Hungary [10], i.e. in less economically developed areas.

Socioeconomic inequalities in smoking habits have been enlarging since the Seventies in most Western countries [4, 20–25], including Italy [8, 13]. However during the last decade the trend is somewhat confusing: indeed in the 3^rd^ millennium in Australia social disadvantage did not increase among current smokers according to two national surveys out of three [26]. Likewise educational inequalities remained stable in Canada according to Reid et al. [5], while they increased according to other studies [27, 28]. In The Netherlands, educational inequalities in smoking prevalence widened in women, but not in men [19]. Similarly in Italy the prevalence of current smokers was lower in women with primary school education in 2000, but this socioeconomic pattern tended to reverse during the last decade [13]. According to the HBSC study [29] absolute differences in daily smoking between secondary school students in vocational or academic tracks increased in Southern Europe (Croatia and Italy) while decreasing in Central Europe (Germany and The Netherlands). In the Minnesota Heart Survey the absolute difference in smoking prevalence between people with higher or lower education peaked during the Nineties and decreased thereafter [3].

Another point under discussion is whether socioeconomic inequalities in smoking habits widen primarily because of increasing differences in smoking initiation [8] or smoking cessation [20] or both [19].

The present work aimed at 1) comparing the effect of education level and occupation on smoking initiation and smoking cessation; 2) investigating whether occupation modified trends in smoking prevalence, separately analyzing the effects on smoking initiation and cessation.

## Methods

### Study design

A cross-sectional survey was performed between 2007 and 2010 in the frame of the GEIRD study (Gene Environment Interactions in Respiratory Diseases) in seven Italian centres: three centres (Torino, Pavia, Verona) were located in Northern Italy, and four (Sassari, Ancona, Terni, Salerno) in Central-Southern Italy [30, 31]. In each centre a sample of about 3,000 subjects, with a male to female ratio of one, was selected from the general population aged 20–44 years, using local health authority registry. Overall 18,357 subjects were administered a screening questionnaire by mail. Non-responders were contacted again, first twice by mail and then by phone, achieving a final response percentage of 57.2% (10,494/18,357). For this reason, in each centre the screening phase lasted about two years, from sample selection to the last phone contact, so that age at interview ranged from 20 to 47 year. A full description of the study design is given at http://www.geird.org.

In a repeated cross-sectional study smoking prevalence observed in GEIRD was compared with prevalence recorded between 1998–2000 in the frame of the Italian Study of Asthma in Young Adults (ISAYA) [15], using data from the four centres participating in both surveys (Turin, Pavia, Verona, Sassari). Of note, ISAYA and GEIRD were multicentre cross-sectional surveys on respiratory diseases, carried out by the same research team on random samples of young adults, using the same design, sampling strategy and questions on smoking habits. In the 4 centres participating in both studies the number of participants was 8931 in the first survey and 5162 in the second one.

### Questionnaire

The screening questionnaire, used in GEIRD [available at http://www.geird.org], was the same questionnaire used in ISAYA [32] with the addition of questions on education level, outdoor exposure, history of asthma, rhinitis, chronic bronchitis and eczema, and life impairment.

Subjects were considered ever smokers if they reported to have smoked at least one cigarette per day or one cigar a week for one year. Ever smokers were divided into ex-smokers, if they had stopped smoking for at least one month, or smokers otherwise. The remaining subjects were considered never smokers. If a subject was suspected, but not deemed, to be an ever smoker due to contradictory responses on smoking habits, he/she was excluded from the analysis. To evaluate cumulative smoke exposure, pack-years were calculated as years of smoking multiplied by the average daily consumption of 20-cigarette packs. Questions on smoking habits had been previously validated in one participating centre (Verona) [33].

### Statistical analysis

Significance of differences in the proportion of current smokers and ex-smokers was investigated by the chi-squared test. As quantitative variables (age at start smoking, cigarettes smoked daily, pack-years) were asymmetrically distributed and presented substantial heteroskedasticity, significance of differences was assessed by non-parametric tests (Wilcoxon-Mann–Whitney rank-sum test or Kruskal-Wallis rank test).

To separately investigate smoking initiation and smoking cessation, two separate logistic regression models were applied to estimate, respectively, 1) the probability of being an ever smoker in the whole sample (initiation ratio), 2) the probability of being an ex-smoker among ever smokers (quit ratio).

In the cross-sectional analyses, comprising data from all the seven centres participating in GEIRD cross-sectional survey, two-level logistic models were used with level-1 units (subjects) nested into level-2 units (GEIRD centres). Gender, age (20–29, 30–39, > = 40 years), education level (primary school, lower secondary school, upper secondary school, university), occupation (clerk, housewife, manager, businessman, free-lancer, workman, unemployed, student, other) and geographic area (Northern vs. Central-Southern Italy) were introduced in the model as explanatory variables, while season of response and type of contact (postal vs. phone) were also considered as potential confounders. To counteract possible selection bias [34], the percentile rank of cumulative response was also computed: in each centre responders were ordered according to response date and were attributed a percentile rank, taking as 100% the total number of eligible subjects in that centre [31]. This variable was then introduced in the model as a measure of promptness to respond. Significance of the interaction between sex and education was also tested. The influences of education and occupation on smoking habits were compared by computing the Akaike Information Criterion (AIC) [35] of models including either education or occupation.

In the repeated cross-sectional analyses, considering the four centres participating in both ISAYA and GEIRD, “centre” was introduced in the models as an explanatory variable along with time period (ISAYA/GEIRD), gender, age, occupation. Season at the interview, type of contact and percentile rank of cumulative response were taken into account as potential confounders. The latter variable was used to counteract possible selection bias, as response percentage had decreased from the first (72.7%) to the second (57.2%) survey and this trend likely affected prevalence estimates [34]. Significance of the interactions between time and either sex, age class, or occupation was also tested to verify whether temporal trends in smoking habits varied as a function of the latter variables.

## Results

### Cross-sectional survey

In the seven centres participating in the GEIRD cross-sectional study, information on smoking habits was available in 10,289 subjects (98%). Of these, current smokers were 2854 (27.7%) and ex-smokers 1662 (16.2%). The prevalence of current smokers was higher in men (32.6%) than in women (23.3%), while the difference in ex-smoker prevalence was smaller (17.4% vs. 15.0% respectively) (Table 1).

**Table 1:** **Smoking habits in 10,289 subjects participating in GEIRD cross-sectional survey in 2007–2010**

	Never smokers	Ex-smokers	Current smokers	***p***value
Sex^b^				p < 0.001^a^
Men	2451 (50%)	852 (17.4%)	1600 (32.6%)	
Women	3320 (61.7%)	810 (15.0%)	1253 (23.3%)	
Age^b^				p < 0.001
20-29 years	1707 (59.8%)	276 (9.7%)	872 (30.5%)	
30-39 years	2525 (56.4%)	759 (17.0%)	1193 (26.6%)	
40-47 years	1520 (51.9%)	627 (21.4%)	782 (26.7%)	
Education^b^				p < 0.001
Primary school	45 (43.2%)	14 (13.5%)	45 (43.3%)	
Lower secondary	763 (40.3%)	374 (19.7%)	757 (40.0%)	
Upper secondary	3110 (56.6%)	887 (16.2%)	1493 (27.2%)	
Degree	1816 (66.2%)	382 (13.9%)	544 (19.8%)	
Occupation^b^				p < 0.001
Clerk	2448 (60.4%)	703 (17.4%)	899 (22.2%)	
Housewife	430 (58.8%)	118 (16.1%)	183 (25.0%)	
Manager	101 (58.7%)	37 (21.5%)	34 (19.8%)	
Businessman	186 (50.0%)	70 (18.8%)	116 (31.2%)	
Free-lancer	692 (54.5%)	201 (15.8%)	377 (29.7%)	
Workman	616 (41.8%)	282 (19.2%)	573 (39.0%)	
Unemployed	309 (46.7%)	93 (14.0%)	260 (39.3%)	
Student	756 (67.5%)	81 (7.2%)	283 (25.3%)	
Retired	18 (56%)	5 (16%)	9 (28%)	
Other job	186 (51.6%)	68 (18.8%)	107 (29.6%)	
Site of residence^b^				p = 0.002
Downtown	1794 (54.9%)	496 (15.2%)	978 (29.9%)	
Suburbs	2285 (56.4%)	662 (16.3%)	1103 (27.2%)	
Countryside	568 (54.4%)	205 (19.6%)	272 (26.0%)	
Geographic area				p = 0.001
Northern Italy	2163 (56.4%)	676 (17.6%)	999 (26.0%)	
Central-Southern	3610 (55.9%)	986 (15.3%)	1855 (28.8%)	

^a^: P values were computed by chi-squared test.

^b^: information on sex, age, education, occupation, site of residence was missing respectively in 3, 28, 59, 48, 1922 subjects. Of note, the Ancona sample (n = 1866) was not asked about their site of residence.

Current smoking slightly decreased with advancing age, while past smoking largely increased as expected. Current smoking was twice as prevalent in people with a primary/secondary school certificate (40-43%) compared with people with an academic degree (20%), and among unemployed and workmen (39%) compared with managers and clerks (20-22%). Conversely ex-smokers were rather rare among people with only primary school license and among students and unemployed, and quite common among managers. The prevalence of current smokers was slightly higher in urban areas and in Central-Southern Italy, while the prevalence of ex-smokers was somewhat higher in rural areas and in Northern Italy (Table 1).

Men smoked 2.7 cigarettes per day more than women, and the number of cigarettes smoked daily increased with advancing age (Table 2). Smokers with lower education had started smoking at an earlier age and consumed more cigarettes per day than smokers with higher education. As a consequence, cumulative smoking exposure at the age of 30 years was nearly doubled in current smokers with only primary school license with respect to those with a university degree. Workmen and unemployed, when current smokers, had started smoking one year earlier, consumed three-four cigarettes per day more than managers, free-lancers and clerks. As a consequence, cumulative smoke exposure at 30 years was lower by about three pack-years in the latter groups (Table 2).

**Figure 1:**
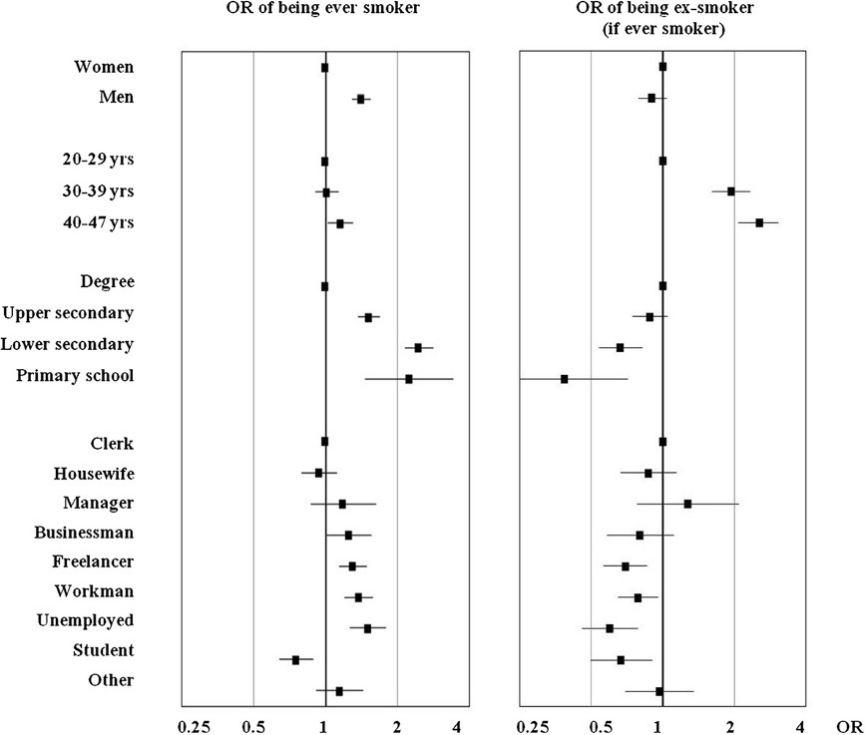
**Analysis of risk factors for smoking initiation and cessation in the GEIRD cross-sectional study, performed in 2007–2010.** Columns are Odds Ratios (ORs), bars are 95% confidence intervals. The ORs of being an ever smoker (to the right) or an ex-smoker if ever smokers (to the left) were computed by two-level logistic regression models, comprising sex, age, education level, occupation, geographic area, season of response, percentile rank of cumulative response and type of contact. Retired (n=32) were not considered in the logistic model.

In multivariable analysis, the odds of being an ever smoker were significantly increased in men with respect to women (p < 0.001), while the odds of being an ex-smoker among ever smokers were similar in both sexes (p = 0.133) (Figure 1). At variance, age strongly affected the process of quitting smoking but exerted only minor influences on the process of starting smoking.

**Table 2:** **Age at start smoking, number of cigarettes smoked daily and pack-years smoked at 30 year in 2854 current smokers retrieved in GEIRD cross-sectional survey, as a function of sex, age, education level, occupation, site of residence and climatic region**

	No. of smokers	Age at start smoking	Cigarettes/day	Pack-years smoked at 30 ^a^
Sex		p = 0.111^b^	p < 0.001	p < 0.001
Men	1600	17.3 ± 3.7 (17, 15–18)	13.8 ± 10.7 (10, 8–20)	9.7 ± 8.3 (8.3, 4.5-13)
Women	1253	17.5 ± 3.8 (17, 15–19)	11.1 ± 6.7 (10, 6–15)	7.7 ± 5.5 (6.5, 3.8-10.5)
Age		p < 0.001	p < 0.001	p = 0.009
20-29 years	872	16.6 ± 2.5 (16, 15–18)	10.4 ± 6.5 (10, 5–15)	-----
30-39 years	1193	17.6 ± 3.7 (17, 15–19)	13.1 ± 7.8 (10, 8–20)	8.5 ± 7.6 (7.5, 4–11.3)
40-47 years	782	17.9 ± 4.7 (17, 15–20)	14.1 ± 8.7 (13, 8–20)	9.3 ± 6.7 (7.8, 4.2-13)
Education		p < 0.001	p < 0.001	p < 0.001
Primary school	45	16.0 ± 4.3 (15.5, 14–18)	17.1 ± 11.4 (15, 10–20)	12.8 ± 10.3 (10.5,5.5-17)
Lower secondary	757	16.5 ± 3.3 (16, 15–18)	15.8 ± 10.6 (15, 10–20)	11.2 ± 8.8 (10, 6–15)
Upper secondary	1493	17.5 ± 3.6 (17, 15–19)	11.6 ± 8.6 (10, 6–20)	8.0 ± 6.4 (7, 3.6-11.3)
Degree	544	18.3 ± 4.2 (18, 16–20)	10.2 ± 6.7 (10, 5–15)	6.6 ± 4.9 (5.6, 3–9.6)
Occupation		p < 0.001	p < 0.001	p < 0.001
Clerk	899	17.9 ± 4.1 (17, 15–19)	11.2 ± 7.1 (10, 5–15)	7.4 ± 5.4 (6, 3–10.5)
Housewife	183	17.4 ± 4.1 (17, 15–19)	12.9 ± 7.4 (12, 10–15)	8.8 ± 6.3 (7.1, 4.9-12)
Manager	34	17.8 ± 5.0 (17, 15–20)	11.6 ± 7.9 (10, 8–15)	8.4 ± 7.6 (6, 4.5-8)
Businessman	116	17.8 ± 3.4 (17, 15–20)	14.4 ± 8.7 (15, 9–20)	9.1 ± 6.3 (8.4, 5–12)
Free-lancer	377	18.0 ± 4.0 (17, 16–20)	11.9 ± 7.0 (10, 6–15)	7.7 ± 5.4 (7, 3.5-11.3)
Workman	573	16.8 ± 3.2 (16, 15–18)	15.5 ± 13.8 (15, 10–20)	10.9 ± 10.2 (9, 5.9-14)
Unemployed	260	16.9 ± 3.6 (16, 15–18)	14.4 ± 8.7 (12, 10–20)	10.8 ± 8.3 (9, 5–14)
Student	283	16.7 ± 2.4 (17, 15–18)	8.8 ± 5.1 (10, 5–10)	7.5 ± 4.5 (8.1, 3.5-11.3)
Retired	9	14.7 ± 3.1 (15, 13–15)	18.0 ± 7.9 (18, 14–25)	14.2 ± 8.7(13.1,7.4-19.4)
Other job	107	16.7 ± 3.7 (16, 15–18)	13.2 ± 8.1 (10, 8–20)	9.8 ± 6.8 (8.1, 5–12.8)
Site of residence^c^		p =0.238	p = 0.089	p = 0.055
Downtown	978	17.5 ± 3.8 (17, 15–19)	12.5 ± 10.0 (10, 7–18)	8.8 ± 8.4 (7.5, 3.9-12)
Suburbs	1103	17.4 ± 3.8 (17, 15–19)	12.7 ± 9.6 (10, 7–18)	8.9 ± 7.0 (7.5, 4–12)
Countryside	272	17.4 ± 4.1 (16, 15–18)	13.4 ± 7.8 (11, 8–20)	9.6 ± 6.3 (8, 5–14)
Geographic area		p =0.474	p =0.241	p =0.697
Northern Italy	999	17.3 ± 3.6 (17, 15–19)	12.7 ± 9.8 (10, 7–16)	8.8 ± 6.6 (7, 3.9-12)
Central-Southern	1855	17.5 ± 3.9 (17, 15–19)	12.3 ± 7.9 (10, 6–17)	8.8 ± 7.7 (7.5, 4.2-12)

Data are expressed as mean ± SD (median, interquartile range).

^a^: Pack-years smoked by 30 yrs were computed by multiplying the average daily consumption of 20-cigarette packs by years from age at starting smoking to 30, in current smokers aged 30 years or more.

^b^: *P* values were computed by Wilcoxon Mann–Whitney rank-sum test or by Kruskal-Wallis rank test.

^c^: the Ancona sample (n = 1866) was not asked about their site of residence.

Education markedly affected both starting and quitting smoking: with respect to people with a university degree, the odds of being an ever smoker were more than doubled in people with a primary or lower secondary school license, while the odds of being an ex-smoker among ever smokers were greatly reduced especially in people with primary school education only (p < 0.001). Starting and quitting smoking were largely affected also by occupation. Unemployed presented the highest initiation ratio and the lowest quit ratio. A similar, although less pronounced, pattern was observed also among workmen and free lancers, who had higher odds of being an ever smoker and lower odds of being an ex-smoker as compared to clerks. The odds of being an ex-smoker was the highest among managers. A model including only education minimized the AIC with respect to a model considering only occupation. The difference was substantial when evaluating risk factors for smoking initiation (13673 vs. 13777) but only minor when addressing smoking cessation (5674 vs. 5678).

A significant quantitative interaction between sex and education level was detected when addressing smoking initiation (p = 0.002), but not when studying smoking cessation (0.782). With respect to men with university education, the odds of being an ever smoker was more than three times higher in men with lower education (Odds Ratio (OR) =3.4, 95% confidence interval (CI) 1.8-6.4 in those with primary school license and OR = 3.1, 2.6-3.8 with lower secondary education), while in women a two-fold increase was recorded (OR = 1.5, 0.8-2.8 and OR = 2.0, 1.6-2.4 respectively).

### Repeated cross-sectional survey

The main socio-demographic characteristics of the two samples, collected in the same four centres in the frame of ISAYA (1998–2000) and GEIRD cross-sectional (2007–10) are presented in Table 3. The second sample was slightly older and presented a slightly higher proportion of women. Smoking prevalence declined in both sexes during the last decade, from 39.6% to 32.3% in men and from 31.0% to 21.6% in women. The decline was very limited in people aged 20–29 years (from 35.5% to 29.0%) and particularly pronounced in people aged 40 years and over (from 36.9% to 24.6%). As regards occupation, smoking prevalence decreased remarkably among managers, businessmen, free-lancers and clerks (respectively from 35.1%, 41.9%, 37.9%, 31.7% to 20.5%, 31.3%, 27.6%, 21.8%), but only slightly among unemployed and students (respectively from 39.1%, 28.4% to 36.1%, 24.1%). Intermediate decreases were recorded among workmen and housewives (respectively from 44.4%, 29.8% to 37.1%, 22.4%).

**Table 3:** **Main socio-demographic characteristics and smoking status of people participating to either ISAYA (1998–2000) or GEIRD (2007–2010) in four centers**

	ISAYA	GEIRD	p-value
	(n = 8931)	(n = 5162)	
Centres			<0.001
Verona	2166 (24.3%)	1746 (33.8%)	
Pavia	2444 (27.4%)	966 (18.7%)	
Turin	2266 (25.4%)	1205 (23.3%)	
Sassari	2055 (23.0%)	1245 (24.1%)	
Sex			<0.001
Men	4439 (49.7%)	2397 (46.4%)	
Women	4492 (50.3%)	2765 (53.6%)	
Occupation			<0.001
Clerk	3444 (38.7%)	2114 (41.3%)	
Housewife	786 (8.8%)	352 (6.9%)	
Manager	151 (1.7%)	90 (1.8%)	
Businessman	268 (3.0%)	153 (3.0%)	
Free-lancer	901 (10.1%)	570 (11.1%)	
Workman	1381 (15.5%)	777 (15.2%)	
Unemployed	564 (6.3%)	316 (6.2%)	
Student	864 (9.7%)	549 (10.7%)	
Retired	42 (0.5%)	20 (0.4%)	
Other job	498 (5.6%)	175 (3.4%)	
Smoking habits			<0.001
Never smokers	4356 (49.0%)	2809 (55.4%)	
Ex-smokers	1404 (15.8%)	915 (18.0%)	
Current smokers	3138 (35.3%)	1347 (26.6%)	
Age (mean ± SD)	33.4 ± 6.8	34.8 ± 7.1	<0.001

The average number of cigarettes (mean ± SD), consumed daily by current smokers, significantly declined in men from 15.3 ± 8.7 (median 15, interquartile range 10–20) to 13.7 ± 8.4 (12, 8–20) (p < 0.001), while this decline was hardly appreciable, if any, in women (from 11.6 ± 7.3 to 10.9 ± 6.9 cigarettes/day, p = 0.117). Age at smoking initiation, instead, did not change from the first survey (17.3 ± 3.3 years) to the second one (17.4 ± 3.9 years) (p = 0.079) in both sexes.

The number of cigarettes smoked daily significantly declined from the first to the second survey among clerks (from 12.8 ± 8.2 to 11.1 ± 7.3 cigarettes/day; p = 0.001) and free-lancers (from 14.5 ± 8.2 to 11.5 ± 6.8 cigarettes/day; p = 0.002). Non-significant decreases of 1–2 cigarettes per day were recorded also among managers, businessmen and students. Of note, smoking intensity did not vary among workmen and unemployed and even tended to increase among housewives, from 11.9 ± 7.2 to 13.5 ± 8.5 cigarettes/day.

Also in multivariable analysis the OR of being an ever smoker decreased from the first to the second survey (Table 4). The declining trend varied as a function of gender (time-sex interaction: p = 0.029), age class (time-age interaction: p < 0.001) and occupation (time-occupation interaction: p = 0.047): the time-related decrease in ever smoking was slightly larger in women than in men, and it was particularly pronounced in people aged > =40 years, while being not significant in people younger than 30 years. As regards occupation, the declining trend was particularly evident in housewives, managers, businessmen and free-lancers, while starting smoking became even more common among unemployed.

**Table 4:** **Analysis of temporal trends and relevant risk factors in smoking initiation and cessation in the four centers participating to both ISAYA (1998–2000) and GEIRD (2007–2010)**

	OR of being ever smoker (95% CI)		OR of being ex-smoker
	N = 10,263		(95% CI) N = 4,468
Time			p < 0.001
ISAYA 1998-2000			1
GEIRD 2007-2010			1.32 (1.18-1.48)
	Interaction sex*time: p = 0.029		
Sex	ISAYA 1998-2000	GEIRD 2007-2010	p = 0.760
Women	1	0.71 (0.65-0.79)	1
Men	1.42 (1.30-1.56)	1.19 (1.07-1.33)	0.98 (0.87-1.10)
	Interaction age*time: p < 0.001		
Age	ISAYA 1998-2000	GEIRD 2007-2010	p < 0.001
20-29 years	1	0.95 (0.83-1.08)	1
30-39 years	1.26 (1.14-1.40)	1.00 (0.88-1.12)	1.93 (1.67-2.23)
40-47 years	1.84 (1.62-2.09)	1.09 (0.95-1.24)	2.47 (2.10-2.89)
	Interaction occupation*time: p = 0.047		
Occupation	ISAYA 1998-2000	GEIRD 2007-2010	p < 0.001
Clerk	1	0.74 (0.66-0.83)	1
Housewife	1.25 (1.06-1.46)	0.75 (0.59-0.94)	1.07 (0.88-1.31)
Manager	1.01 (0.73-1.41)	0.68 (0.44-1.04)	0.95 (0.64-1.40)
Businessman	1.55 (1.19-2.01)	1.05 (0.75-1.46)	0.87 (0.66-1.16)
Free-lancer	1.14 (0.98-1.32)	0.78 (0.65-0.94)	0.76 (0.64-0.91)
Workman	1.49 (1.31-1.70)	1.27 (1.08-1.49)	0.75 (0.64-0.87)
Unemployed	1.11 (0.92-1.33)	1.24 (0.98-1.57)	0.61 (0.48-0.78)
Student	0.67 (0.56-0.79)	0.60 (0.49-0.73)	0.70 (0.54-0.91)
Other job	1.47 (1.21-1.79)	1.13 (0.83-1.54)	0.96 (0.76-1.22)

Odds Ratios (ORs) and 95% confidence intervals (CI) were obtained by logistic regression models, comprising time (ISAYA/GEIRD), centre, sex, age, occupation, season of response, percentile rank of cumulative response and type of contact.

The column dealing with the trend of smoking initiation (being ever smoker) was split in two as there were significant interactions between time and the main predictors (sex, age, occupation). The column dealing with smoking cessation (being ex-smoker) was not split as no significant interaction emerged.

The OR of being an ex-smoker among ever smokers increased by one third from the first to the second survey, and the decline was not significantly modified by gender, age or occupation. As already shown in the previous cross-sectional analysis, the odds of being an ex-smoker increased with advancing age, were the lowest in unemployed, and were not affected by gender.

## Discussion

The main findings of the present study are:

1. During the last decade the prevalence of current smoking has significantly declined in Italy in both sexes, due to both a decrease in smoking initiation and a simultaneous increase in smoking cessation. The decreasing trend in smoking initiation was slightly more pronounced in women than in men; as regards age, the decline was nearly absent in people aged 20–29 years and particularly pronounced in people aged 40–47 years. Age at smoking initiation has not changed during the last decade, while the number of cigarettes smoked daily has declined in male smokers, but not in females.
2. As regards occupation, the declining trend in smoking initiation was particularly evident in housewives, managers, businessmen and free-lancers, while starting smoking became even more common among unemployed. Instead the decreasing trend in smoking cessation was not affected by occupation.
3. As a consequence, nowadays in Italy initiation ratios are the highest in lower socio-economic classes, i.e. in people with only primary or secondary school education, and in unemployed and workmen. Conversely these classes also present the lowest quit ratios. Education proved to be a stronger predictor of smoking habits than occupation. The inverse association between smoking and education level, although significant in both sexes, is stronger in men than in women.
4. Current smokers belonging to the lower socioeconomic classes present a greater cumulative smoking exposure than their counterparts in the higher socioeconomic classes, resulting from both an earlier age at starting smoking and a greater number of cigarettes consumed daily. Cumulative smoking exposure at the age of 30 years is nearly doubled in current smokers with only primary school license with respect to those with a university degree.

It should be reminded that the Italian centres participating in ISAYA or GEIRD were not chosen randomly, but on the presence of experienced research teams willing to carry out the survey. The prevalence estimates of current and past smoking, however, are in line with those reported by other surveys carried out on Italian national samples by the Italian National Statistical Institute (ISTAT) [13, 36] or DOXA-Mario Negri-Istituto Superiore di Sanità [1, 37]. At variance, these surveys reported that the decreasing trend in smoking among Italian women had levelled off in the last decade, which is opposite to the results of the present survey.

In the 3^rd^ millennium socioeconomic inequalities in smoking habits are still widening in Italy in both sexes [13], at variance with other Western countries where this trend seems to have levelled off [3] or to continue only in women [19]. In Italy the prevalence of current smoking is decreasing among higher socioeconomic classes but not among lower classes, and this pattern mainly reflects a parallel pattern in smoking initiation, in agreement with another Italian study [8]. Accordingly, the absolute difference in daily smoking between secondary school students in vocational or academic tracks has increased by 8.8% from 2002 to 2010 [29]. Moreover, number of cigarettes smoked daily was higher in socially disadvantaged smokers, as reported in the current literature [5, 19, 38], and this inequality has been enlarging during the last decade, as observed in Dutch men [19].

In the present study education proved to be a more important determinant of smoking initiation and cessation than occupation. Of note, education and occupation were strongly related: people with primary school licence were mainly workmen, housewives and unemployed, while people with university degree were mainly clerks and freelancers. While other surveys, performed in Southern Europe as well [1, 7], did not find any difference in smoking initiation between women with low or high education, in the present survey as well as in another Italian study [13] smoking initiation was inversely related to education level also in women, but the association was less strong than in men.

The present research has some limitations. First of all, response percentage decreased from the first (72.7%) to the second (57.2%) survey, and this trend could affect prevalence estimates, as ex-smokers are early responders while current smokers are late responders in epidemiological surveys [34]. However, to counteract possible selection bias, risk estimates were adjusted for percentile rank of cumulative response and type of contact (postal vs. phone). Second, in the present survey the definition for ex-smokers was not very rigorous, as an abstinence of just one month was required, according to the European Community Respiratory Health Survey (ECRHS) questionnaire [32]. Third, reports of smoking status were not verified with biochemical methods. Nevertheless, in one participating centre (Verona) a good agreement had been found (Cohen’s k = 0.93) between self-reported smoking consumption and serum cotinine levels [33]. Fourth, recall bias could have affected self-reporting of age at smoking initiation or cessation, as individuals tend to attribute the onset of a habit to an age closer to the time of interview than the true age at onset [39]. However, no recall bias had been detected in a similar survey, the Italian branch of ECRHS: age at start smoking, reported by 313 subjects interviewed twice 8.6 years apart, was only 0.01 ± 2.01 years lower in the second interview with respect to the first one.

Data collection in the present study ended in 2010, before the peak of economic crisis in Italy. Indeed in GEIRD cross-sectional the proportion of unemployed was 6.5%, which is compatible with national Italian data in 2008 (6.7%), while it has peaked at 13.6% in the first trimester 2014 [http://dati.istat.it/Index.aspx?DataSetCode=DCCV_TAXDISOCCU]. It can be expected that such large increase in unemployment has remarkably affected smoking prevalence: for instance, in the United States during the economic crisis the expected decrease in smokers among employed was counterbalanced by a largely unexpected increase in smokers among unemployed [40].

## Conclusions

In conclusion, in Italy the prevalence of current smoking has decreased during the last decade in both sexes, thanks to both a decrease in smoking initiation and an increase in smoking cessation. However, the decreasing trend, while pronounced in the highest socioeconomic classes, has not started yet among the lowest classes. This divergent pattern mainly reflects a different trend in smoking initiation and is amplified by enlarging difference in smoking intensity. Health inequalities related to tobacco smoke are particularly large when socioeconomic status is evaluated by considering education rather than occupation. As a consequence, anti-smoking campaigns should focus on socioeconomically disadvantaged teenagers.

### Ethical approval

Ethics approval was obtained in each centre involved in GEIRD from the appropriate ethics committee (Comitato Etico dell’Azienda Ospedaliero-Universitaria Ospedali Riuniti di Ancona; Comitato di Bioetica della Fondazione IRCCS Policlinico San Matteo di Pavia; Comitato Etico dell’Azienda Sanitaria Locale SA/2 di Salerno; Comitato di Bioetica dell’Azienda Sanitaria Locale di Sassari; Comitato Etico delle Aziende Sanitarie dell’Umbria di Perugia; Comitato Etico dell’Azienda Sanitaria Locale TO/2 di Torino; Comitato Etico per la Sperimentazione dell’Azienda Ospedaliera Istituti Ospitalieri di Verona). All participants were fully informed about all aspects of the research project and consented to complete and return the questionnaire.
