# Correction to: Socioeconomic inequalities in smoking habits are still increasing in Italy

**DOI:** 10.1186/s12889-017-4829-z

**Published:** 2017-10-17

**Authors:** Giuseppe Verlato, Simone Accordini, Giang Nguyen, Pierpaolo Marchetti, Lucia Cazzoletti, Marcello Ferrari, Leonardo Antonicelli, Francesco Attena, Valeria Bellisario, Roberto Bono, Lamberto Briziarelli, Lucio Casali, Angelo Guido Corsico, Alessandro Fois, Maria Grazia Panico, Pavilio Piccioni, Pietro Pirina, Simona Villani, Gabriele Nicolini, Roberto de Marco

**Affiliations:** 1 Unit of Epidemiology and Medical Statistics, Department of Public Health and Community Medicine, University of Verona, Verona, Italy; 2 Department of Medicine, University of Verona, Verona, Italy; 3 Allergy Unit, Ospedali Riuniti di Ancona, Ancona, Italy; 4 Department of Public, Clinical and Preventive Medicine, II University of Naples, Naples, Italy; 5 Department of Public Health and Pediatrics, University of Turin, Turin, Italy; 6 Department of Hygiene, University of Perugia, Perugia, Italy; 7 Department of Internal Medicine, Section of Respiratory Disease, University of Perugia, Perugia, Italy; 8 Institute of Respiratory Diseases, IRCCS San Matteo, Pavia, Italy; 9 Institute of Respiratory Diseases, University of Sassari, Sassari, Italy; 10 Unit of Epidemiology, ASL, 2 Salerno, Salerno Italy; 11 Unit of Respiratory Medicine, National Health Service (CPA-ASL TO2), Turin, Italy; 12 Department of Public Health, Experimental and Legal Medicine, University of Pavia, Pavia, Italy; 13 Corporate Clinical Development, Chiesi Farmaceutici S.p.A, Parma, Italy; 14 Sezione di Epidemiologia e Statisca Medica, Istituti Biologici 2B, Strada Le Grazie 8, 37134 Verona, Italy

## Correction

After publication of the article [1], it has been brought to our attention that the full funding acknowledgement is missing from the original article. It should also include the following –.

“The GEIRD project was funded also by the Italian Ministry of Health (Ministero della Salute).”

Hence the correct version of the list of funding institutions (Page 9, “Competing Interests”) is the following:

“The GEIRD project was funded by the Cariverona Foundation (Verona, Italy), by the Italian Ministry of Health (Ministero della Salute) and by the Italian Ministry of Education, Universities and Research (MIUR). The funding sources had no involvement in the study design, in the collection, analysis and interpretation of data, in the writing of the report, and in the decision to submit the paper for publication.”
